# Comparative renal outcomes of matched cohorts of patients with type 2 diabetes receiving SGLT2 inhibitors or GLP-1 receptor agonists under routine care

**DOI:** 10.1007/s00125-024-06251-z

**Published:** 2024-08-23

**Authors:** Gian Paolo Fadini, Enrico Longato, Mario Luca Morieri, Enzo Bonora, Agostino Consoli, Bruno Fattor, Mauro Rigato, Federica Turchi, Stefano Del Prato, Angelo Avogaro, Anna Solini, on behalf of DARWIN-Renal Study Investigators

**Affiliations:** 1 Division of Metabolic Diseases, Department of Medicine, University of Padova, Padova, Italy; 2 Laboratory of Experimental Diabetology, Veneto Institute of Molecular Medicine, Padova, Italy; 3 Department of Information Engineering, University of Padova, Padova, Italy; 4 Section of Endocrinology, Diabetes and Metabolism, Department of Medicine, University of Verona, Verona, Italy; 5 Department of Medicine and Aging Sciences (DMSI) and Center for Advanced Studies and Technology (CAST), University G. D’Annunzio of Chieti-Pescara, Chieti, Italy; 6 Diabetology Service, Azienda Sanitaria dell’Alto Adige, Bolzano, Italy; 7 Diabetology Service, Department of Medicine, Azienda ULSS 2 Marca Trevigiana, Treviso, Italy; 8 Metabolic Disease and Diabetology Unit, IRCCS INRCA, Ancona, Italy; 9 Department of Clinical & Experimental Medicine, University of Pisa and Sant’Anna School of Advanced Studies, Pisa, Italy; 10 Department of Surgical, Medical, Molecular and Critical Area Pathology, University of Pisa, Pisa, Italy

**Keywords:** Kidney disease, Obesity, Pharmacoepidemiology, Real-world, Type 2 diabetes

## Abstract

**Aims/hypothesis:**

We compared the effects of sodium–glucose cotransporter 2 (SGLT2) inhibitors (SGLT2i) and glucagon-like peptide-1 receptor agonists (GLP-1RA) on renal outcomes in individuals with type 2 diabetes, focusing on the changes in eGFR and albuminuria.

**Methods:**

This was a multicentre retrospective observational study on new users of diabetes medications. Participant characteristics were assessed before and after propensity score matching. The primary endpoint, change in eGFR, was analysed using mixed-effects models. Secondary endpoints included categorical eGFR-based outcomes and changes in albuminuria. Subgroup and sensitivity analyses were performed to assess robustness of the findings.

**Results:**

After matching, 5701 participants/group were included. Participants were predominantly male, aged 61 years, with a 10 year duration of diabetes, a baseline HbA_1c_ of 64 mmol/mol (8.0%) and BMI of 33 kg/m^2^. Chronic kidney disease (CKD) was present in 23% of participants. During a median of 2.1 years, from a baseline of 87 ml/min per 1.73 m^2^, eGFR remained higher in the SGLT2i group compared with the GLP-1RA group throughout the observation period by 1.2 ml/min per 1.73 m^2^. No differences were detected in albuminuria change. The SGLT2i group exhibited lower rates of worsening CKD class and favourable changes in BP compared with the GLP-1RA group, despite lesser HbA_1c_ decline. SGLT2i also reduced eGFR decline better than GLP-1RA in participants without baseline CKD.

**Conclusions/interpretation:**

In individuals with type 2 diabetes, treatment with SGLT2i was associated with better preservation of renal function compared with GLP-1RA, as evidenced by slower decline in eGFR. These findings reinforce SGLT2i as preferred agents for renal protection in this patient population.

**Graphical Abstract:**

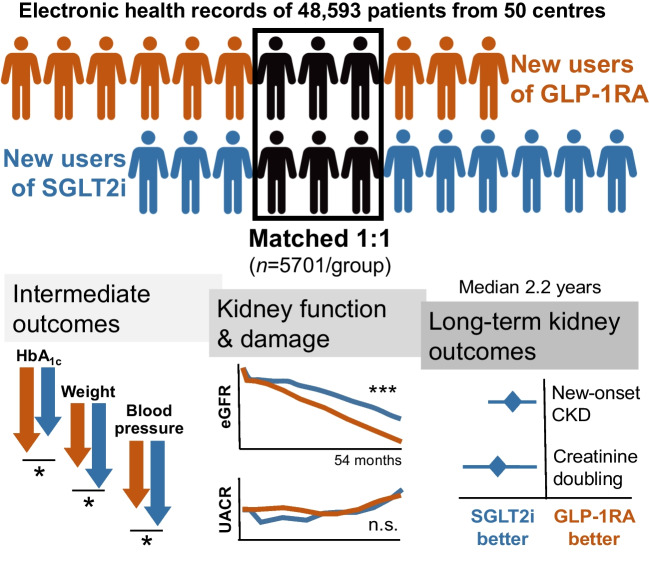

**Supplementary Information:**

The online version of this article (10.1007/s00125-024-06251-z) contains peer-reviewed but unedited supplementary material.


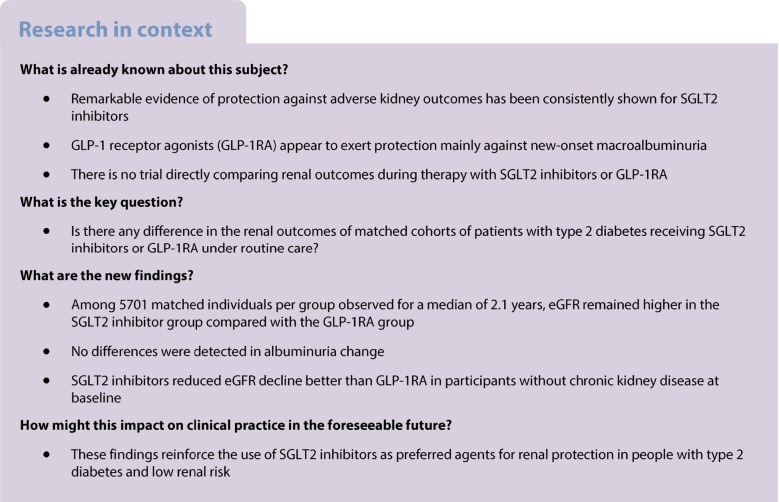


## Introduction

Diabetic kidney disease (DKD) is the leading cause of end-stage kidney disease (ESKD) globally [1] and it poses a substantial burden on society and healthcare systems [2, 3]. The impact of chronic kidney disease (CKD) in type 2 diabetes extends beyond renal dysfunction, encompassing a spectrum of systemic complications and comorbidities. The intricate interplay between diabetes and CKD amplifies the risk of adverse outcomes, necessitating early intervention and targeted management strategies.

Treatment for DKD aims to mitigate renal dysfunction, attenuate disease progression and reduce the risk of complications. Multifaceted management includes lifestyle modification, pharmacotherapy and targeted interventions to optimise glucose, BP, weight and lipid control. Renin–angiotensin system (RAS) blockers remain a cornerstone therapy for DKD, offering benefits beyond BP control [4]. On top of that, several landmark clinical trials have provided robust evidence supporting the renal benefits of sodium–glucose cotransporter 2 (SGLT2) inhibitors (SGLT2i), revolutionising the management of DKD. Secondary analysis of cardiovascular outcome trials (CVOTs) have reported remarkable improvements in all kidney outcomes [5–7]. Such evidence has been corroborated by trials showing nephroprotective effects of SGLT2i in individuals with CKD of any origin, with or without type 2 diabetes [8, 9]. Based on the compelling evidence from clinical trials, international guidelines endorse the use of SGLT2i as preferred agents for reducing the risk of kidney disease progression in individuals with type 2 diabetes [10]. Although less prominently, glucagon-like peptide-1 receptor agonists (GLP-1RA) can also attenuate renal dysfunction in type 2 diabetes. CVOTs found significant reductions in incident or worsening nephropathy with GLP-1RA compared with placebo [11]. While this was mainly due to a protection from new-onset macroalbuminuria, there was some evidence of slower loss of kidney function [12]. The FLOW study was dedicated to exploring the effects of semaglutide against the progression of kidney damage in individuals with type 2 diabetes and albuminuric CKD [13]: during a median of 3.4 years, semaglutide improved a composite outcome of kidney failure; ≥50% reduction in the eGFR; or death from kidney or cardiovascular causes [14]. The mechanisms whereby both SGLT2i and GLP-1RA can preserve kidney function encompass the improvements in glycaemia, BP and body weight. Yet, SGLT2i are believed to exert greater haemodynamic activity, while GLP-1RA have a more prominent anti-inflammatory action [15].

No randomised trial has directly compared the renal effects of SGLT2i with those of GLP-1RA, nor is any such trial planned. Indirect comparisons through network meta-analyses conclude that SGLT2i ensure a greater protection than GLP-1RA against DKD progression [16–18]. Real-world studies on this comparison provide mixed results. Some studies have reported better preservation of renal function with SGLT2i than with GLP-1RA [19–21], while others have reported similar kidney outcomes with either drug class [22, 23].

In view of this knowledge gap, we devised and performed a multicentre retrospective observational study on clinical-level data, with the aim of comparing the long-term kidney outcomes of patients who initiated SGLT2i or GLP-1RA under routine care.

## Methods

### Study design and objectives

DARWIN-Renal was a multicentre retrospective study conducted by the Italian Diabetes Society at 50 specialist care centres in Italy, with the primary aim of analysing renal outcomes associated with the use of dapagliflozin. The study rationale and design have been described before [24], and the analytical framework was extended to all SGLT2i as a class [25]. The protocol complied with the declaration of Helsinki and was approved by the Ethics Committee of all participating centres. According to the national regulation on retrospective studies using anonymised data, patients’ informed consent was waived. The study was funded by the Italian Diabetes Society and partly supported by an unrestricted grant from AstraZeneca. This report conforms with the STROBE checklist, as modified for the comparison of matched cohorts.

We herein report a predefined analysis comparing patients initiating any SGLT2i vs any GLP-1RA.

### Cohort definition

We selected two groups of patients: (1) those who initiated any SGLT2i (dapagliflozin, empagliflozin, canagliflozin, ertugliflozin) between 1 January 2015 and 30 September 2020; and (2) those who initiated any GLP-1RA (exenatide twice daily; exenatide, liraglutide, lixisenatide, dulaglutide, semaglutide once weekly) in the same time period. The index date was the day when the SGLT2i or GLP-1RA was prescribed for the first time. Patients could be included if they were aged 18–80 years, had type 2 diabetes for at least one year and had available information on renal outcomes. The exclusion criteria were as follows: other forms of diabetes; therapy with SGLT2i or GLP-1RA in the prior 12 months; simultaneous initiation of SGLT2i and GLP-1RA; concomitant initiation of insulin; CKD stage V; or dialysis.

### Data collection

The set of variables recorded from the electronic chart for each patient is described elsewhere [25, 26]. Briefly, at the index date (with a grace period of −90 days) and at each follow-up time point, we recorded demographics (sex was recorded as reported in the patients’ ID documents; race/ethnicity were not available in the database), anthropometrics, BP, laboratory data, presence or absence of chronic complications, and background therapy for diabetes and for the control of cardiovascular risk factors. We also collected pre-index-date eGFR values to compute the baseline eGFR slope.

### Endpoints

The primary endpoint was the change in eGFR, according to the CKD-EPI equation [27]. Secondary outcomes were as follows: total and chronic (from 6 months on) eGFR slopes; changes in urinary albumin/creatinine ratio (UACR), HbA_1c_, body weight and BP; new-onset CKD (defined as the occurrence of two eGFR values <60 ml/min per 1.73 m^2^ at least 90 days apart, among those who had a baseline eGFR >60 ml/min per 1.73 m^2^); worsening in CKD class (stage I eGFR ≥90 ml/min per 1.73 m^2^; stage II 60–90 ml/min per 1.73 m^2^; stage IIIa 45–60 ml/min per 1.73 m^2^; stage IIIb 30–45 ml/min per 1.73 m^2^; stage VI 15–30; stage V <15 ml/min per 1.73 m^2^); sustained loss of kidney function (defined as an eGFR reduction of 40% or greater relative to baseline value); sustained doubling of serum creatinine (equal to a reduction in eGFR of 57% or greater relative to baseline value); ESKD (defined as a confirmed eGFR <15 ml/min per 1.73 m^2^ on at least two occasions at least 90 days apart); and initiation of dialysis.

### Sample size

For the comparison of eGFR change (primary endpoint) between the two groups, it was estimated that a sample size of 1184 individuals per group was needed to detect a difference of 2 ml/min per 1.73 m^2^ with power 0.9 and α 0.05.

### Statistical analysis

Continuous variables are presented as mean (SD), while categorical variables are reported as *n* (%). Non-normal variables were log-transformed before analysis with parametric tests (log-transformed variables are shown in their original unit of measure). Comparisons between two groups were performed using the Wilcoxon–Mann–Whitney test or the χ^2^ test, as appropriate.

The change over time in continuous variables was compared between the two groups using the mixed model for repeated measures (MMRM). eGFR, UACR (log_10_), HbA_1c_, body weight and BP were used as the dependent variables. Treatment group, time, the group by time interaction, and baseline values were entered as fixed effects. The heterogeneous compound symmetry was chosen as the variance structure. The output of the MMRM were the marginal means in each group and the mean difference between groups, and their SEs. Rates of occurrence of categorical outcomes were compared between the two groups using the Cox proportional hazards model, reporting HRs and 95% CIs. The proportional hazards assumption was verified by visual inspection and Schoenfeld residuals.

To yield comparable groups, we performed propensity score matching (PSM) of patients who initiated SGLT2i or GLP-1RA. Propensity scores were calculated with a logistic regression where treatment was the dependent variable; covariates are listed in Table 1, chosen using the modified disjunctive cause criterion [28]. Individuals in the two groups were matched 1:1 with a calliper of 0.1 pooled SD using nearest neighbour method without replacement. Between-group balance before and after PSM was evaluated by calculating the standardised mean difference (SMD). Success of matching was defined as SMD<0.1 for all variables listed in Table 1. Because PSM requires a complete dataset, we performed multiple imputation by chained equations (MICE) to obtain ten imputed datasets. Imputation was performed on the same variables used for PSM, without a priori constraints and setting the maximum number of iterations to 20. All analyses were run on each of the ten datasets and results were then pooled. Imputation had the sole scope of enabling the calculation of propensity score for matching and was not intended to overcome the missingness in outcome data. Indeed, post-index date outcome variables were not imputed and, for each outcome, individuals with missing follow-up data were excluded. Persistence of a good balance in subgroups of the matched populations was verified and variables that were consistently imbalanced (SMD>0.1) in ≥50% of the imputed datasets were entered as covariates in the MMRM or survival analyses. As done before [25], the comparison between matched cohorts was performed with an unpaired approach because the balance of clinical characteristics was reached only between groups and not within pairs of matched individuals. This approach has been shown to yield results that do not significantly deviate from those of the paired analysis [25].

**Table 1:** Characteristics of the ITT population (patients in the first imputed dataset)

Characteristic	Before PSM			After PSM		
	SGLT2i	GLP−1RA	SMD	SGLT2i	GLP−1RA	SMD
Number	14435	6226		5705	5705	
Demographics						
Sex, male, *n* (%)	8988 (62.3)	3691 (59.3)	0.06	3462 (60.7)	3408 (59.7)	0.02
Age, years	61.4 (8.9)	61.0 (9.5)	0.04	60.9 (9.2)	60.8 (9.5)	0.02
Diabetes duration, years	11.8 (8.6)	10.1 (8.0)	0.20	9.9 (7.8)	9.9 (8.0)	<0.01
Anthropometrics						
Weight, kg	88.8 (18.1)	94.3 (18.9)	0.30	92.2 (18.9)	92.8 (17.8)	0.03
Height, cm	167.3 (9.7)	167.5 (9.8)	0.03	167.7 (9.8)	167.6 (9.8)	<0.01
BMI, kg/m^2^	31.7 (5.8)	33.6 (6.2)	0.31	32.8 (6.2)	33.0 (5.7)	0.04
Waist, cm	109.0 (13.4)	112.8 (13.7)	0.28	111.3 (14.1)	111.8 (12.8)	0.04
Risk factors and laboratory measurements						
Systolic BP, mmHg	137.8 (19.0)	137.5 (18.7)	0.02	137.2 (18.4)	137.3 (18.5)	<0.01
Diastolic BP, mmHg	79.2 (10.2)	80.1 (10.1)	0.08	79.9 (10.3)	80.0 (10.2)	<0.01
Fasting plasma glucose, mmol/l mg/dl	9.6 (3.2) 172.6 (58.2)	9.1 (2.8) 163.0 (49.3)	0.17	9.1 (2.8) 163.7 (50.4)	9.1(5.5) 163.7 (49.6)	<0.01
HbA_1c_, mmol/mol	68 (12)	64 (10)	0.27	64 (10)	64 (10)	<0.01
HbA_1c_, %	8.4 (1.5)	8.0 (1.3)		8.0 (1.3)	8.0 (1.3)	
Total cholesterol, mmol/l	4.5 (1.1)	4.5 (1.1)	<0.01	4.6 (1.1)	4.5 (1.1)	0.01
Total cholesterol, mg/dl	172.3 (43.5)	172.7 (43.3)		173.4 (42.6)	172.8 (43.5)	
HDL-cholesterol, mmol/l	1.2 (0.4)	1.2 (0.4)	0.01	1.2 (0.4)	1.2 (0.4)	0.01
HDL-cholesterol, mg/dl	46.6 (14.3)	46.8 (13.8)		47.0 (14.0)	46.8 (13.9)	
LDL-cholesterol, mmol/l	2.5 (1.0)	2.5 (1.0)	0.03	2.5 (0.9)	2.5 (0.9)	<0.01
LDL-cholesterol, mg/dl	93.4 (36.2)	94.5 (36.1)		94.9 (35.8)	94.6 (36.0)	
Triglycerides, mmol/l	1.9 (1.4)	1.8 (1.3)	0.04	1.8 (1.3)	1.8 (1.3)	<0.01
Triglycerides, mg/dl	167.5 (125.2)	162.8 (118.8)		163.6 (114.7)	162.5 (119.9)	
Baseline eGFR, ml/min per 1.73 m^2^	86.7 (16.6)	84.1 (19.9)	0.15	85.9 (17.1)	85.8 (18.5)	<0.01
eGFR at month −12, ml/min per 1.73 m^2^	87.7 (18.1)	84.1 (20.0)	0.13	86.8 (18.0)	86.6 (18.8)	0.01
AER, mg/g	68.8 (360.5)	70.4 (336.8)	<0.01	62.4 (360.4)	63.5 (302.0)	<0.01
eGFR slope, ml/min per 1.73 m^2^ per year^a^	−0.5 (17.0)	−0.7 (15.9)	0.01	−0.4 (15.1)	−0.6 (16.1)	0.01
Complications, *n* (%)						
CKD stage III or higher	879 (6.1)	815 (13.1)	0.26	479 (8.4)	518 (9.1)	0.02
Pathological albuminuria	2354 (16.3)	1016 (16.3)	<0.01	877 (15.4)	889 (15.6)	<0.01
Diabetic retinopathy	2749 (19.0)	820 (13.2)	0.16	696 (12.2)	718 (12.6)	0.01
Diabetic macular oedema	395 (2.7)	105 (1.7)	0.07	101 (1.8)	95 (1.7)	<0.01
Stroke / TIA	216 (1.5)	84 (1.3)	0.01	67 (1.2)	73 (1.3)	<0.01
Carotid atherosclerosis	2876 (19.9)	1210 (19.4)	0.01	989 (17.3)	1095 (19.2)	0.05
Ischaemic heart disease	2108 (14.6)	645 (10.4)	0.13	620 (10.9)	598 (10.5)	0.01
Left ventricular hypertrophy	1122 (7.8)	446 (7.2)	0.02	398 (7.0)	393 (6.9)	<0.01
Heart failure	487 (3.4)	145 (2.3)	0.06	148 (2.6)	137 (2.4)	0.01
Any site revascularisation	1489 (10.3)	487 (7.8)	0.08	496 (8.7)	450 (7.9)	0.03
Microvascular complications	5518 (38.2)	2279 (36.6)	0.03	1890 (33.1)	1919 (33.6)	0.01
Macrovascular complications	5150 (35.7)	1933 (31.0)	0.10	1769 (31.0)	1755 (30.8)	<0.01
Established CVD	2514 (17.4)	808 (13.0)	0.12	757 (13.3)	743 (13.0)	<0.01
Glucose-lowering medications, *n* (%)						
Metformin	11487 (79.6)	5109 (82.1)	0.06	4782 (83.8)	4768 (83.6)	<0.01
Sulfonylurea / repaglinide	901 (6.2)	779 (12.5)	0.23	636 (11.1)	661 (11.6)	0.01
Pioglitazone	234 (1.6)	291 (4.7)	0.19	178 (3.1)	205 (3.6)	0.03
Acarbose	87 (0.6)	29 (0.5)	0.02	26 (0.5)	27 (0.5)	<0.01
Bolus insulin	4248 (29.4)	189 (3.0)	0.67	195 (3.4)	189 (3.3)	<0.01
Basal insulin	6307 (43.7)	1473 (23.7)	0.42	1303 (22.8)	1327 (23.3)	<0.01
Other medications, *n* (%)						
Statins	8281 (57.4)	3339 (53.6)	0.08	3090 (54.2)	3043 (53.3)	0.02
Antiplatelet agent	5894 (40.8)	2243 (36.0)	0.10	2074 (36.4)	2022 (35.4)	0.02
RAS blocker	8559 (59.3)	3685 (59.2)	<0.01	3335 (58.5)	3332 (58.4)	<0.01
Beta blocker	4364 (30.2)	1776 (28.5)	0.04	1629 (28.6)	1613 (28.3)	<0.01
Calcium-channel inhibitor	3074 (21.3)	1408 (22.6)	0.03	1278 (22.4)	1258 (22.1)	<0.01
Diuretic	4189 (29.0)	2052 (33.0)	0.09	1783 (31.3)	1777 (31.1)	<0.01
Anticoagulant	353 (2.4)	155 (2.5)	<0.01	137 (2.4)	128 (2.2)	0.01

Data are presented as mean (STD) or as *n* (%), as appropriate

Absolute SMD is shown for each variable as a measure of between-group balance

^a^eGFR according to the CKD-EPI equation

TIA, transient ischaemic attack

The primary analysis was conducted in the intention-to-treat (ITT) population, comprising all new users of SGLT2i or GLP-1RA who had at least one eGFR value post-index date, censored at event occurrence or last observation. We performed a sensitivity analysis on the on-treatment population, censoring patients at the time of index drug discontinuation, the event or last observation, whichever occurred first. Drug discontinuation was defined as the first follow-up visit at which the drug was no longer being prescribed. A further, modified on-treatment analysis incorporated censoring when patients in the SGLT2i group initiated GLP-1RA and vice versa.

The primary endpoint was re-examined in subgroups of patients based on pre-specified clinical characteristics at baseline. Patients were divided into strata and the mean between-group difference in eGFR was calculated in each stratum and compared across strata. Interaction *p* values were reported as nominal and adjusted with Bonferroni correction.

We performed a sensitivity analysis excluding patients with CKD at baseline (eGFR <60 ml/min per 1.73 m^2^ or UACR >30 mg/g), which required that multiple imputation and PSM be repeated in the new datasets.

The conventional statistical significance threshold of 0.05 was used, without a hierarchical testing, except that secondary endpoints were analysed only when significance on the primary endpoint was met. The analyses were run in R 4.2.2 (R Development Core Team; https://www.r-project.org/), using the MatchIt, mice, glmmTMB, stats and survival packages.

## Results

### Patient disposition and characteristics

**Fig. 1:**
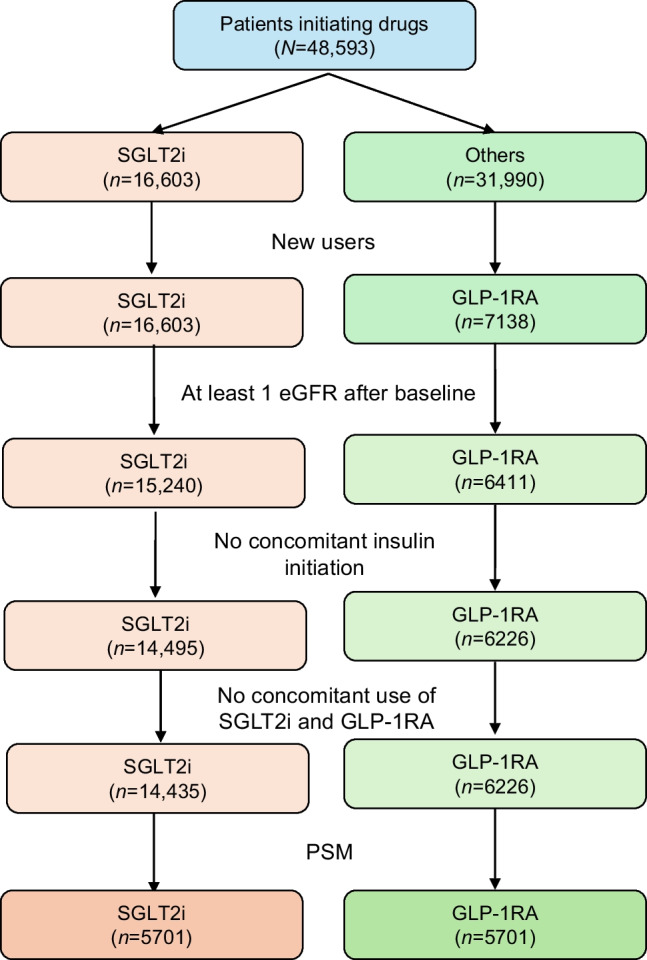
Study flow chart

From a population of 48,593 patients, after applying exclusion criteria, we identified 14,435 new users of SGLT2i and 6226 new users of GLP-1RA who had at least one post-baseline eGFR and who were not initiated with insulin treatment or were not receiving concomitant treatment with an SGLT2i and GLP-1RA (Fig. 1). Before matching, new users of SGLT2i had longer diabetes duration, lower BMI, higher fasting plasma glucose, HbA_1c_ and eGFR, and a greater prevalence of retinopathy and CVD (Table 1). Concomitant glucose-lowering medications were also different between the groups before matching, with a lower proportion of individuals on oral medications and more on insulin in the SGLT2i group.

After matching, the analysis included a mean of 5701 individuals per group, with small differences across the ten imputed dataset: the first imputed dataset shown in Table 1 contained 5705 (range 5684–5719) individuals per group. All analyses were performed in each imputed dataset and then pooled. The matched populations were 60% men, aged 61 years and with a diabetes duration of 10 years. Mean BMI was 33 kg/m^2^, baseline HbA_1c_ 64 mmol/mol (8.0%) and eGFR 86 ml/min per 1.73 m^2^. Only 8–9% had an eGFR <60 ml/min per 1.73 m^2^ but ~15% had UACR >30 mg/g (23% had CKD). While one-third of the patients had any microangiopathy and one-third had any macroangiopathy, only 13% had established CVD. The most common concomitant glucose-lowering medications were metformin (84%) and basal insulin (23%). These characteristics are representative of the T2D population seen in diabetes specialist care in Italy.

The newly initiated SGLT2i were distributed as follows: dapagliflozin (52.8%; mean daily dose 9.9 mg); empagliflozin (38.6%; mean daily dose 15.6 mg); canagliflozin (8.5%; mean daily dose 172 mg); and ertugliflozin (<0.1%; mean daily dose 15 mg). The newly initiated GLP-1RA were distributed as follows: dulaglutide (52.3%; mean weekly dose 1.34 mg); liraglutide (30.8%; mean daily dose 1.35 mg); exenatide (10.7%; 2 mg weekly dose); semaglutide (3.8%; mean weekly dose 0.6 mg); and lixisenatide (2.3%; mean daily dose 10 mg).

### Change in eGFR

**Fig. 2:**
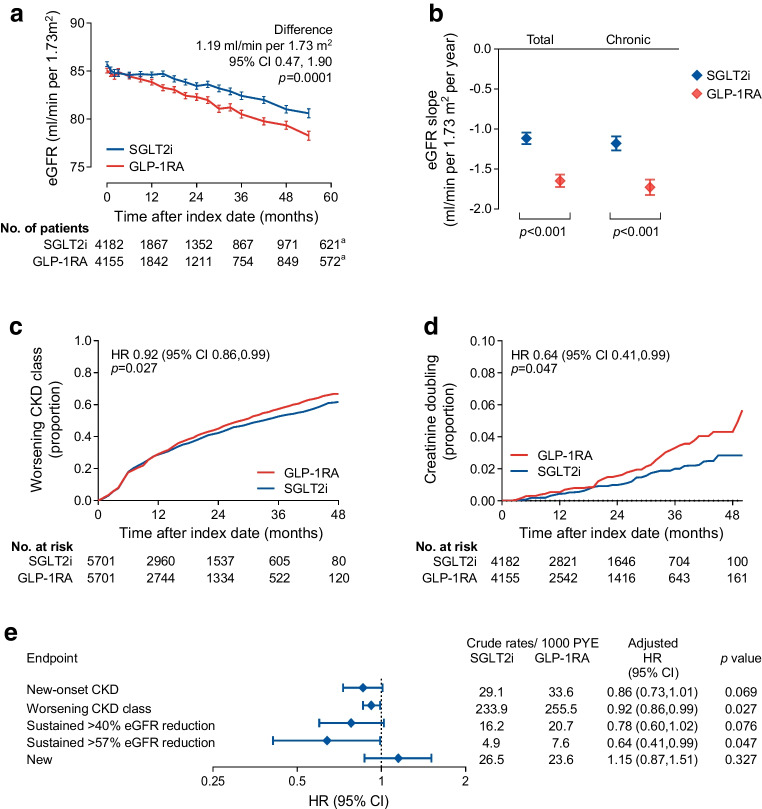
Major kidney outcomes. (**a**) Change in eGFR (primary outcome) in the two groups (^a^54 months). The table shows the number of patients contributing with values to the model. (**b**) Total and chronic eGFR slopes. (**c**, **d**) Kaplan–Meier curves for the worsening of CKD class (**c**) and creatinine doubling (≥57% reduction in eGFR) (**d**). The tables show the number of patients at risk. (**e**) Forest plot of kidney outcomes in the ITT population. Tabular results are presented as crude rates in each group, HRs and 95% CIs along with their *p* values

The analysis was based on a median of 5 (IQR 3–9) eGFR values for each patient in both groups. The median (IQR) follow-up time was 2.1 years (1.1–334). The observation was closed at 54 months because the residual sample size dropped below 10% after that time point. The eGFR slope from months −12 to 0 was −0.8 and −0.9 ml/min per 1.73 m^2^ per year in the SGLT2i group and GLP-1RA group, respectively. The two groups were matched for eGFR at baseline and at month −12 (Table 1). After the index date, the eGFR remained significantly higher in the SGLT2i group for the entire duration of observation, with a difference of 1.19 (95% CI 0.47, 1.90) ml/min per 1.73 m^2^ (*p*=0.0001; Fig. 2a).

The total post-index-date eGFR slope was less negative in the SGLT2i group vs the GLP-1RA group by 0.5 ml/min per 1.73 m^2^ per year (95% CI 0.3, 0.8): −1.1 vs −1.6 ml/min per 1.73 m^2^ per year; (*p*<0.001; Fig. 2b). Results for the chronic slope, calculated from 6 months onwards, were almost identical (*p*<0.001).

### Loss of kidney function

The hazard of worsening of CKD class was significantly lower in the SGLT2i group than in the GLP-1RA group (HR 0.92 [95% CI 0.86, 0.99]; *p*=0.027; Fig. 2c). In addition, the rate of creatinine doubling (≥57% eGFR loss) was lower in the SGLT2i group than in the GLP-1RA group (HR 0.64 [95% CI 0.41, 0.99]; *p*=0.047; Fig. 2d). The other eGFR-based categorical outcomes were all in favour of SGLT2i, although the differences were not statistically significant (Fig. 2e). In all analyses, the events of ESKD and dialysis were too low to be compared between groups.

### Change in albuminuria

This analysis was conducted on a mean of 4819 individuals across the ten imputed datasets with available follow-up values for UACR. In five imputed datasets, the concomitant use of sulfonylurea was imbalanced between groups and was adjusted. The analysis was based on a median of four UACR values per individual in both groups. UACR consistently declined at 6–24 months in the SGLT2i group but the change in UACR over time was similar for the two groups (ESM Fig. 1a). There was also no difference in the rates of new-onset macroalbuminuria (ESM Fig. 1b).

### Intermediate endpoints

**Fig. 3:**
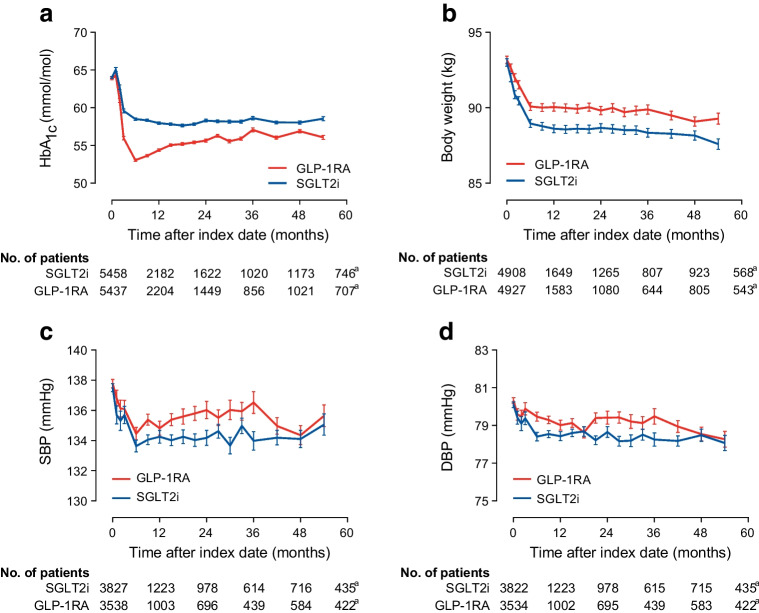
Intermediate endpoints. The change over time in HbA_1c_ (**a**), body weight (**b**), systolic BP (**c**) and diastolic BP (**d**) is shown for the two groups and compared using the MMRM. The tables show the number of patients contributing with values at each time point of the model. ^a^54 months. DBP, diastolic BP; SBP, systolic BP

HbA_1c_ declined to a greater extent with GLP-1RA than with SGLT2i, by 1.8 mmol/mol (0.2%; *p*<0.0001; Fig. 3a). On the other hand, body weight declined to a greater extent in the SGLT2i group, by 1.3 kg (*p*=0.0008; Fig. 3b). A greater improvement in systolic BP was seen with SGLT2i than with GLP-1RA by 1.1 mmHg (*p*=0.001; Fig. 3c); the same was true of diastolic BP (−0.6 mmHg; *p*=0.0006; Fig. 3d).

### Subgroup analyses

**Fig. 4:**
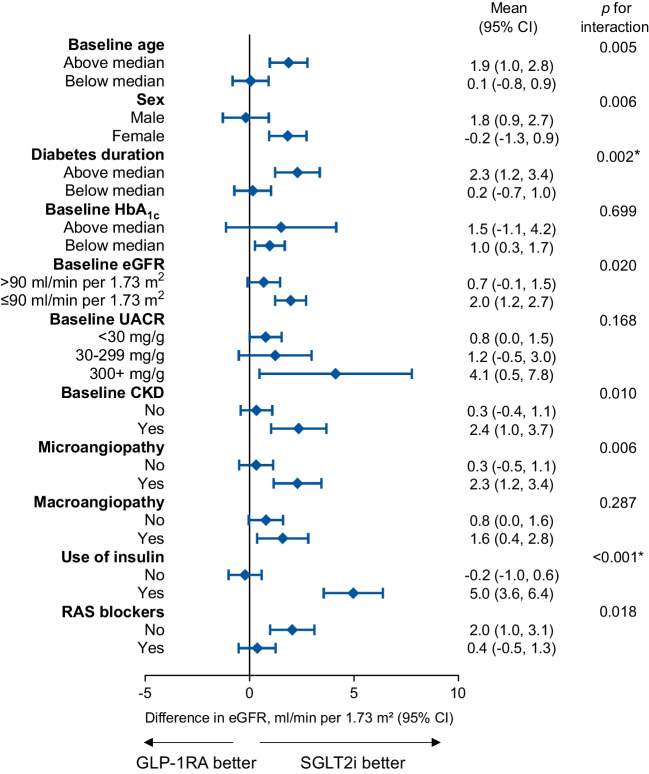
Subgroup analysis. The primary endpoint (change in eGFR) was computed for each stratum of the initial population and compared between the SGLT2i and GLP-1RA groups. Nominal *p* values are shown (*significant after adjusting with Bonferroni correction)

The primary outcome was re-examined in strata of the ITT population defined by key baseline characteristics. After correction for multiple testing, new users of SGLT2i displayed less decline in eGFR than new users of GLP-1RA, especially if they had a longer disease duration or were on insulin (Fig. 4). There was no difference by sex.

The on-treatment population included a mean of 5624 matched patients per group (ESM Table 1). For the primary endpoint, during a median observation of 1.7 years (IQR 0.9, 2.9) in each group, eGFR declined less with SGLT2i than with GLP-1RA by 1.45 ml/min per 1.73 m^2^ (95% CI 0.72, 2.19; *p*=0.0001). The rates of new-onset CKD (HR 0.83 [95% CI 0.69, 0.99]; *p*=0.041) and worsening of CKD class (HR 0.93 [95% CI 0.86, 0.99]; *p*=0.037) remained in favour of SGLT2i, whereas the other eGFR-based categorical outcomes were not significantly different between groups (ESM Fig. 2). Likewise, no differences were detected in the change in albuminuria, albuminuria class or rates of new-onset macroalbuminuria. Results for intermediate endpoints were almost superimposable to those in the ITT analysis (ESM Table 2). There were 1.8% of individuals in the SGLT2i group who initiated GLP-1RA or vice versa after the index date. Additional censoring at these events did not meaningfully modify the primary outcome (difference in eGFR 1.35 ml/min per 1.73 m^2^ [95% CI 0.62, 2.09]; *p*=0.0004).

We also performed a sensitivity analysis excluding patients with CKD at baseline; this included a mean of 4416 matched individuals per group, all having eGFR>60 ml/min per 1.73 m^2^ and UACR<30 mg/g at baseline (ESM Table 3). During a median of 1.8 years (IQR 1.0–3.1), eGFR declined less with SGLT2i than with GLP-1RA by 1.12 ml/min per 1.73 m^2^ (95% CI 0.43, 1.81; *p*=0.0015; ESM Fig. 3a). The rate of worsening CKD class was significantly lower in the SGLT2i group (HR 0.89 [95% CI 0.82, 0.96]; *p*=0.0017; ESM Fig. 3b), whereas other eGFR-based categorical outcomes did not differ significantly (ESM Fig. 3e, ESM Table 2). The change in albuminuria was similar between groups (ESM Fig. 3c) but the rate of new-onset macroalbuminuria was greater in the SGLT2i than in the GLP-1RA group (HR 2.03; 95% CI 1.14–3.63; *p*=0.00; ESM Fig. 3d). When a confirmatory value was requested for macroalbuminuria, the rates were not significantly different between groups (HR 1.56 [95% CI 0.87, 2.80]). Intermediate endpoints showed similar differences as in the primary analysis (ESM Table 2).

## Discussion

Among matched patients with type 2 diabetes, initiation of SGLT2i was associated with better renal outcomes than initiation of GLP-1RA. New users of SGLT2i displayed less decline in eGFR over time, yielding a 1.2 ml/min per 1.73 m^2^ higher value during a median observation of about 2 years and a slope that was less negative by 0.5 ml/min per 1.73 m^2^ per year than in new users of GLP-1RA. SGLT2i also provided protection against the worsening of CKD class and the doubling of serum creatinine (ITT population) or new-onset CKD (on-treatment population). These findings confirm that SGLT2i are more effective in providing protection against the loss of kidney function.

It is remarkable that these results were obtained in a population of patients with an overall preserved eGFR (mean 86 ml/min per 1.73 m^2^). However, 23% of the entire population had CKD defined by eGFR or albuminuria criteria. To rule out the possibility that this small CKD subgroup drove the different kidney outcomes, we performed a secondary analysis excluding all individuals with CKD before matching. Starting from an eGFR of ~90 ml/min per 1.73 m^2^, SGLT2i initiators displayed slower eGFR decline and a lesser degree of worsening of eGFR class. In this population, however, new users of GLP-1RA had lower rates of new-onset macroalbuminuria.

Our findings need to be interpreted in the light of recent observational studies. In a large US multi-database study, a composite renal outcome (eGFR decline ≥50%, ESKD, or all-cause mortality) had similar rates in the SGLT2i and GLP-1RA cohorts [23]. Similar results were obtained in a Swedish study examining a composite renal outcome that included albuminuria [22]. On the other hand, in a population-based study in Hong Kong, users of SGLT2i had a slower eGFR decline than users of GLP-1RAs, by about 0.7–0.8 ml/min per 1.73 m^2^ per year, along with a lower rate of the composite kidney outcome [20]. Another retrospective study from Japan found that SGLT2i use was associated with a slower eGFR decline when compared with GLP-1RA use [19]. The inconsistency of these results may be due to inclusion of macroalbuminuria in the composite outcome but other methodological aspects are crucial and our study has notable strengths [24]. The data source was highly homogeneous and all patients were followed in the same specialist setting, with uniform access to care and universal public coverage. The availability of several clinical laboratory variables ensured that PSM generated similar cohorts, emulating the target trial, because matched patients had the same probability of treatment with either drug class. Matching on variables that define severity and disease stage, along with the new-user design, reasonably ruled out time-lag bias and immortal time bias [29]. With an observation time reaching 54 months, this is one of longest studies on this comparison. This is important because a follow-up of at least 2 years is recommended for the calculation of reliable eGFR slopes [30]. However, slopes can produce unrealistically positive values due to the recovery after a transient drop in eGFR after SGLT2i initiation [31]. This is why we elected to analyse eGFR change over time using the MMRM, which makes no assumption on the shape of eGFR curves, automatically handles missing data at individual time points, and limits the constrains on the availability of post-index date values. Another notable advantage of our study was the analysis of albuminuria change and the availability of intermediate endpoints, for interpreting the primary results. The similar change in albuminuria between SGLT2i and GLP-1RA well aligns with the notion that GLP-1RA are active against new-onset macroalbuminuria and provides robust study credibility [32]. Yet, among the individuals without CKD, rates of new-onset macroalbuminuria were in favour of GLP-1RA. This finding does not align with trial data [33] or prior observational studies [20]. Speculatively, the finding may be driven by false-positives events in the SGLT2i group due to genitourinary tract infections, as it was attenuated when a confirmatory UACR value in the macroalbuminuric range was requested. SGLT2i were significantly less effective in reducing HbA_1c_ than GLP-1RA, suggesting that protection against eGFR decline may not directly rely on glycaemic control. Conversely, the protection against the rise in albuminuria provided by GLP-1RA may be mediated at least in part by the better glycaemic control [34] and by the anti-inflammatory effects of such agents [35]. Use of SGLT2i was associated with greater improvements in BP, underscoring their prominent haemodynamic effect [15, 36]. Consistent with a network metanalysis of randomised trials [37], body weight reduction was greater in the SGLT2i group. However, semaglutide (the most potent weight-reducing GLP-1RA) accounted for a minority of the GLP-1RA cohort, and not all patients on GLP-1RA reached the highest licenced dose that exerts the greatest effect on body weight. Additionally, the relevance of the difference in weight reduction between the two groups remains unclear, because there is no evidence that weight loss mediates the effects of GLP-1RA on renal outcomes [38].

Subgroup analysis highlights that the superiority of SGLT2i over GLP-1RA was more marked in individuals with features of advanced type 2 diabetes. However, given the non-randomised nature of the study, the between-group balance is not guaranteed across all strata of the population and would require repeating the matching procedure for each stratum.

Drop-in GLP-1RA in the SGLT2i group or vice versa was a rare event because, during most of the study period, the National Health System did not reimburse the SGLT2i/GLP-1RA combination. The complementary mode of action of the two classes warrant considering the combination approach, which may optimise renal protection in type 2 diabetes [26]. Results of the FLOW trial have now provided solid evidence that GLP-1RA (namely semaglutide) can slow the progression of kidney disease in people with albuminuric DKD [14]. Future studies will be needed to explore whether the SGLT2i/GLP-1RA combination is more effective than SGLT2i alone for preventing adverse kidney outcomes [39].

Limitations of our study mainly pertain to its observational non-randomised design. First, we cannot exclude residual confounding due to unmeasured factors. The between-group differences before matching reflect a typical channelling of SGLT2i to patients at a more advanced disease stage, while GLP-1RA are used more in individuals with obesity. Despite matching for several clinical laboratory variables, it is impossible to nullify such selection bias and residual differences may be attributable to neglected or occult factors, including socioeconomic ones [40]. Second, because the study was conducted in the diabetes specialist care setting, results may not be extrapolated to other setting, such as primary care or nephrology. Given that baseline eGFR was ~85 ml/min per 1.73 m^2^ and a minority of patients had CKD at baseline, our findings are poorly relevant to people with type 2 diabetes and established DKD. A dedicated real-world study will be needed to compare the effectiveness of SGLT2i vs GLP-1RA in this specific population. In addition, although results in the on-treatment population strongly supported the ITT analysis, we had no information on adherence to treatment or on the reasons for drug discontinuation, including side effects. Finally, we had no information on competing events, including major adverse cardiovascular events (MACE) and heart failure. Under the assumption that both SGLT2i and GLP-1RA delay mortality of people with type 2 diabetes [41], this should not have a significant effect on the estimation of kidney outcomes.

In conclusion, our study reveals that among matched individuals with type 2 diabetes followed by Italian diabetes specialists, initiation of an SGLT2i was associated with less decline in eGFR over time, with higher eGFR values and less negative eGFR slopes compared with GLP-1RA initiation. These findings support a broad benefit of SGLT2i in preserving kidney function. Our study underscores the potential of SGLT2i as preferred therapeutic agents for renal protection in patients with type 2 diabetes, although the potential for a sequential combination with a GLP-1RA remains to be ascertained.

## Supplementary Information

Below is the link to the electronic supplementary material.

ESM (PDF 464 KB)

## Data Availability

Restrictions apply to the availability of crude data used for this study. Aggregated data are available upon reasonable request via email to the corresponding author.

## Abbreviations

CKD: Chronic kidney disease
CVOT: Cardiovascular outcome trial
DKD: Diabetic kidney disease
ESKD: End-stage kidney disease
GLP-1RA: Glucagon-like peptide-1 receptor agonist(s)
ITT: Intention-to-treat
MMRM: Mixed model for repeated measures
PSM: Propensity score matching
RAS: Renin–angiotensin system
SGLT2: Sodium–glucose cotransporter 2
SGLT2i: SGLT2 inhibitor(s)
SMD: Standardised mean difference
UACR: Urinary albumin/creatinine ratio
