# Supplementary material for: Comparative renal outcomes of matched cohorts of patients with type 2 diabetes receiving SGLT2 inhibitors or GLP-1 receptor agonists under routine care
Source: Diabetologia. 2024 Aug 23;67(11):2585–97. doi: 10.1007/s00125-024-06251-z (PMC11519175; doi:10.1007/s00125-024-06251-z)

Electronic Suppementary Material (ESM)

ESM Table of contents

Content	*Page*
DARWIN-Renal study Investigators	2
ESM Table 1. Characteristics of the on-treatment population	4
ESM Table 2. Study endpoints in the various populations	6
ESM Table 3. Characteristics of the population without baseline CKD	7
ESM Figure 1. Change in albuminuria in the primary ITT analysis	9
ESM Figure 2. Categorical outcomes in the OT population	10
ESM Figure 3. Main results in the population without baseline CKD	11

DARWIN-Renal study Investigators

ABRUZZO. Maria Pompea Antonia Baldassarre, Gloria Formoso, Agostino Consoli (Endocrinology, Diabetes and Metabolism, University Chieti-Pescara). Sara Morgante, Antonella Zugaro, Marco Giorgio Baroni (Diabetes and Andrology Unit, San Salvatore Hospital, University of L'Aquila).

CALABRIA. Francesco Andreozzi (Department of Medical and Surgical Sciences, University Magna Graecia of Catanzaro, Catanzaro).

CAMPANIA. Adriano Gatti (Diabetology Service, ASL Napoli1). Stefano De Riu (Diabetology Unit ASL Napol1 Centro). Andrea Del Buono (Diabetology Unit, ASL Caserta).

EMILIA ROMAGNA. Raffaella Aldigeri, Riccardo Bonadonna (Division of Endocrinology and Metabolic Diseases, Azienda Ospedaliera-Universitaria di Parma, Department of Medicine and Surgery, University of Parma), Alessandra Dei Cas (Division of Nutritional and Metabolic Sciences, Azienda Ospedaliero-Universitaria di Parma, University of Parma), Angela Vazzana, Monica Antonini, Valentina Moretti (Division of Endocrinology and Metabolic Diseases, Azienda Ospedaliera-Universitaria di Parma).

FRIULI VENEZIA GIULIA. Patrizia Li Volsi (Section of Endocrinology and Metabolism, Azienda Sanitaria Friuli Occidentale). Miranda Cesare, Giorgio Zanette (Section of Endocrinology and Metabolism, Pordenone Hospital, Azienda Sanitaria Friuli Orientale).

LAZIO. Silvia Carletti, Paola D'Angelo (Diabetology Unit, Sandro Pertini Hospital, ASL Roma2). Gaetano Leto, Frida Leonetti (Diabetology Unit Latina, Department of Medical-Surgical Sciences and Biotechnologies, Sapienza University, Rome, Italy). Luca D'Onofrio, Ernesto Maddaloni, Raffaella Buzzetti (Diabetology Unit, University of Rome "La Sapienza"). Simona Frontoni (Unit of Endocrinology, Diabetes and Metabolism, S. Giovanni Calibita Fatebenefratelli Hospital, Department of Systems Medicine, University of Rome Tor Vergata, Rome, Italy). Maria Gisella Cavallo, Ilaria Barchetta (Diabetology Unit, Department of Experimental Medicine, Sapienza University, Rome, Italy). Susanna Morano, Tiziana Filardi (University of Rome "La Sapienza", Dept. of Experimental Medicine, Unit of Diabetes and Complications, Clinica Medica V, Azienda Policlinico Umberto I, Roma). Umberto Capece, Andrea Giaccari (Endocrinology and Diabetology, IRCCS Agostino Gemelli University Hospital Foundation, Department of Translational Medicine and Surgery, Catholic University of the Sacred Heart, Rome).

LOMBARDIA. Antonio C. Bossi (Unit of Diabetology, Humanitas Gavazzeni Institute, Bergamo). Giancarla Meregalli (Unit of Diabetology and Metabolic Diseases, Azienda Socio-Sanitaria Territoriale Bergamo Ovest, Treviglio, Bergamo). Fabrizio Querci (Diabetology Unit, Alzano Lombardo Hospital). Alessia Gaglio, Veronica Resi, Emanuela Orsi (Diabetes Unit, Foundation IRCCS Cà Granda Ospedale Maggiore Policlinico, Milan). Stefano Fazion (Diabetology and Metabolic Disease, ASST Mantova). Ivano G. Franzetti (Endocrinology and Diabetology Unit ASST Valle Olona). Cesare Berra (Diabetology and Endocrinology Unit, IRCCS Multimedica, Milan).

MARCHE. Silvia Manfrini (Diabetology Unit, Senigallia). Gabriella Garrapa, Giulio Lucarelli, Lara Riccialdelli (Diabetology Unit, Fano). Elena Tortato (Diabetology and Metabolic Disease, INRCA Ancona).

PIEMONTE. Marco Zavattaro, Gianluca Aimaretti (Division of Endocrinology, Maggiore della Carità Hospital, Department of Translational Medicine, University of Piemonte Orientale, Novara). Franco Cavalot (Diabetes and Metabolic Diseases Unit, San Luigi Gonzaga University Hospital, Turin). Guglielmo Beccuti, Fabio Broglio (Unit of Diabetology and Metabolism, Department of Medical Sciences, University of Turin, 10123 Torino, Italy.).

TRENTINO-ALTO ADIGE. Bruno Fattor (Diabetology Service, Bolzano Hospital).

PUGLIA. Giuliana Cazzetta (Diabetology Unit Tricase (Lecce)). Olga Lamacchia (Department of Medical and Surgical Sciences, University of Foggia). Anna Rauseo, Salvatore De Cosmo (Fondazione IRCCS Casa Sollievo della Sofferenza, San Giovanni Rotondo, Italy).

SARDEGNA. Rosella Cau, Mariangela Ghiani (UO diabetologia Quartu SE, ASL 8 Cagliari).

SICILIA. Antonino Di Benedetto (Diabetology Unit, University Hospital G. Martino, Messina). Antonino Di Pino, Salvatore Piro, Francesco Purrello (Internal Medicine, Garibaldi Nesima Hospital, Department of Clinical and Experimental Medicine, University of Catania). Lucia Frittitta, Agostino Milluzzo (Center for Diabetes and Obesity, Garibaldi Nesima Hospital, Department of Clinical and Experimental Medicine, University of Catania). Giuseppina Russo (Metabolic Disease and Internal Medicine, University Hospital of Messina).

TOSCANA. Anna Solini (Department of Surgical, Medical, Molecular and Critical Area Pathology, University of Pisa). Monia Garofolo, Giuseppe Penno, Stefano Del Prato (Department of Clinical and Experimental Medicine, University of Pisa, Pisa). Roberto Anichini (Diabetes Unit, Area Pistoiese USL Toscana Centro).

VENETO. Gian Paolo Fadini, Angelo Avogaro (Department of Medicine, University of Padova). Lucia Gottardo (Unit of Hypertension and Endocrine-Metabolic-Angiologic Disease, AULSS3 Venice). Mauro Rigato, Agostino Paccagnella (Diabetology Units, Conegliano and Treviso). Marco Strazzabosco (Endocrine, Metabolic and Nutrition Disease, Vicenza Hospital). Massimo Cigolini, Enzo Bonora (Division of Endocrinology, Diabetes and Metabolic Diseases, Department of Medicine, University of Verona).

ESM Table 1. Characteristics of the on-treatment population. Characteristics of patients in the first imputed dataset. Absolute standardized mean difference (SMD) is shown for each variable as a measure of between-group balance.

	Before PSM			After PSM		
	**SGLT2i**	**GLP-1RA**	**SMD**	**SGLT2i**	**GLP-1RA**	**SMD**
**Number**	14282	6153		5643	5643	
**Demographics**						
Sex male, %	8918 (62.4)	3656 (59.4)	0.06	3457 (61.3)	3395 (60.2)	0.02
Age, years	61.4 (8.9)	61.0 (9.5)	0.04	60.9 (9.1)	60.9 (9.4)	<0.01
Diabetes duration, years	11.8 (8.6)	10.1 (8.0)	0.20	9.8 (7.9)	10.0 (8.0)	0.02
**Anthropometrics**						
Weight, kg	88.9 (18.1)	94.4 (18.9)	0.30	92.4 (19.1)	93.0 (17.8)	0.03
Height, cm	167.3 (9.7)	167.6 (9.8)	0.02	167.7 (9.9)	167.6 (9.8)	0.01
Body mass index, kg/m2	31.7 (5.9)	33.6 (6.2)	0.31	32.9 (6.2)	33.1 (5.8)	0.04
Waist, cm	109.0 (13.4)	112.9 (13.7)	0.29	111.3 (13.9)	111.9 (12.9)	0.05
**Risk factors and laboratory**						
Systolic blood pressure, mm Hg	137.6 (18.9)	137.5 (18.8)	<0.01	137.2 (18.7)	137.4 (18.7)	<0.01
Diastolic blood pressure, mm Hg	79.1 (10.1)	80.0 (10.3)	0.09	80.1 (10.2)	80.0 (10.2)	<0.01
Fasting plasma glucose, mg/dl	172.9 (58.3)	163.6 (49.8)	0.17	164.4 (52.0)	164.1 (50.1)	<0.01
HbA1c, mmol/mol<br>%	68.0 (12.1)<br>8.4 (1.5)	64.0 (10.4)<br>8.0 (1.3)	0.27	64.0 (11.2)<br>8.0 (1.4)	64.0 (10.4)<br>8.0 (1.3)	0.01
Total cholesterol, mmol/l<br>mg/dl	4.5 (1.2)<br>172.3 (43.8)	4.5 (1.1)<br>172.8 (43.4)	0.01	4.5 (1.1)<br>172.5 (42.1)	4.5 (1.1)<br>172.8 (43.5)	<0.01
HDL cholesterol, mmol/l<br>mg/dl	1.2 (0.4)<br>46.6 (14.1)	1.2 (0.4)<br>46.7 (13.5)	<0.01	1.2 (0.4)<br>46.7 (13.7)	1.2 (0.4)<br>46.8 (13.5)	<0.01
LDL cholesterol, mmol/l<br>mg/dl	2.5 (1.0)<br>93.5 (36.5)	2.5 (1.0)<br>94.7 (36.4)	0.03	2.5 (0.9)<br>94.5 (35.9)	2.5 (1.0)<br>94.6 (36.4)	<0.01
Triglycerides, mmol/l<br>mg/dl	1.9 (1.4)<br>167.7 (128.6)	1.8 (1.3)<br>162.6 (118.0)	0.04	1.8 (1.2)<br>162.6 (109.8)	1.8 (1.3)<br>162.3 (119.9)	<0.01
eGFR (ml/min/1.73 m^2)	86.7 (16.6)	83.9 (20.0)	0.16	86.1 (17.1)	85.7 (18.4)	0.02
Albumin excretion rate, mg/g	66.8 (341.9)	68.1 (328.6)	<0.01	63.2 (364.9)	64.8 (310.0)	<0.01
eGFR slope (ml/min/1.73 m^2/year)	-0.3 (17.5)	-0.6 (19.3)	0.02	-0.6 (21.8)	-0.4 (19.7)	<0.01
**Complications**						
Chronic kidney disease, %	878 (6.1)	815 (13.2)	0.26	472 (8.4)	502 (8.9)	0.02
Pathologic albuminuria, %	2332 (16.3)	1008 (16.4)	<0.01	875 (15.5)	902 (16.0)	0.01
Diabetic Retinopathy; %	2708 (19.0)	812 (13.2)	0.15	704 (12.5)	717 (12.7)	<0.01
Diabetic macular edema, %	386 (2.7)	104 (1.7)	0.07	89 (1.6)	93 (1.6)	<0.01
Stroke / TIA; %	214 (1.5)	83 (1.3)	0.01	57 (1.0)	72 (1.3)	0.03
Carotid atherosclerosis, %	2848 (19.9)	1198 (19.5)	0.01	990 (17.5)	1085 (19.2)	0.04
Ischemic heart disease; %	2088 (14.6)	642 (10.4)	0.12	619 (11.0)	604 (10.7)	<0.01
Left ventricular hypertrophy, %	1103 (7.7)	437 (7.1)	0.02	400 (7.1)	391 (6.9)	<0.01
Heart failure, %	478 (3.3)	142 (2.3)	0.06	155 (2.7)	132 (2.3)	0.03
Any site revascolarization, %	1476 (10.3)	484 (7.9)	0.08	461 (8.2)	448 (7.9)	<0.01
Microvascular complications, %	5450 (38.2)	2260 (36.7)	0.03	1868 (33.1)	1899 (33.7)	0.01
Macrovascular complications, %	5097 (35.7)	1911 (31.1)	0.10	1745 (30.9)	1746 (30.9)	<0.01
Established CVD, %	2491 (17.4)	803 (13.1)	0.12	747 (13.2)	747 (13.2)	<0.01
**Glucose lowering medications**						
Metformin, %	11366 (79.6)	5049 (82.1)	0.06	4719 (83.6)	4719 (83.6)	<0.01
Sulphonylurea / repaglinide, %	883 (6.2)	759 (12.3)	0.23	603 (10.7)	641 (11.4)	0.02

Pioglitazone, %	231 (1.6)	287 (4.7)	0.19	179 (3.2)	206 (3.7)	0.03
Acarbose, %	87 (0.6)	29 (0.5)	0.02	29 (0.5)	27 (0.5)	<0.01
Bolus insulin, %	4206 (29.4)	188 (3.1)	0.67	189 (3.3)	188 (3.3)	<0.01
Basal insulin, %	6231 (43.6)	1462 (23.8)	0.42	1294 (22.9)	1307 (23.2)	<0.01
**Other medications**						
Statins, %	8203 (57.4)	3312 (53.8)	0.07	3014 (53.4)	3035 (53.8)	<0.01
Anti-platelet agents, %	5831 (40.8)	2218 (36.0)	0.10	1996 (35.4)	2002 (35.5)	<0.01
RAS blockers, %	8478 (59.4)	3650 (59.3)	<0.01	3302 (58.5)	3318 (58.8)	<0.01
Beta blockers, %	4325 (30.3)	1765 (28.7)	0.03	1617 (28.7)	1593 (28.2)	<0.01
Calcium channel inhibitors, %	3048 (21.3)	1398 (22.7)	0.03	1249 (22.1)	1245 (22.1)	<0.01
Diuretics, %	4140 (29.0)	2035 (33.1)	0.09	1735 (30.7)	1789 (31.7)	0.02
Anticoagulants, %	349 (2.4)	153 (2.5)	<0.01	144 (2.6)	125 (2.2)	0.02

Abbreviations: eGFR, estimated glomerular filtration rate according to the CKD-EPI equation. CKD, chronic kidney disease. TIA, transient ischemic attack. CVD cardiovascular disease. RAS, renin angiotensin system.

ESM Table 2. Study endpoints in the various populations.

	Entire population		Without baseline CKD	
	ITT	OT	ITT	OT
N/group	5701	5624	4416	4354
**Primary outcome**				
Change in eGFR, ml/min/1.73 m^2	1.19 (0.47; 1.90)	1.45 (0.72; 2.19)	1.12 (0.43; 1.81)	1.12 (0.42; 1.82)
**Secondary outcomes**				
Difference in total eGFR slope, ml/min/1.73 m^2/year	0.53 (0.30; 0.76)	0.54 (0.28; 0.80)	0.52 (0.26; 0.77)	0.58 (0.29; 0.87)
Difference in chronic eGFR slope, ml/min/1.73 m^2/year	0.55 (0.28; 0.81)	0.67 (0.34; 1.00)	0.48 (0.17; 0.79)	0.67 (0.30; 1.03)
Change in UACR, mg/g	-22.18 (-52.34; 7.98)	-19.29 (-47.92; 9.35)	2.97 (-1.14; 7.07)	2.47 (-1.58; 6.52)
New-onset CKD, HR (95% C.I.)	0.86 (0.73; 1.01)	0.83 (0.69; 0.99)	0.84 (0.69; 1.03)	0.83 (0.66; 1.05)
Worsening CKD class, HR (95% C.I.)	0.92 (0.86; 0.99)	0.93 (0.86; 0.99)	0.89 (0.82; 0.96)	0.90 (0.83; 0.98)
Sustained ≥40% eGFR reduction, HR (95% C.I.)	0.78 (0.60; 1.02)	0.83 (0.62; 1.11)	0.79 (0.57; 1.10)	0.83 (0.59; 1.17)
Sustained ≥57% eGFR reduction, HR (95% C.I.)	0.64 (0.41; 0.99)	0.65 (0.39; 1.09)	0.64 (0.35; 1.19)	0.71 (0.39; 1.27)
New-onset macroalbuminuria, HR (95% C.I.)	1.15 (0.87; 1.51)	1.05 (0.76; 1.46)	2.03 (1.14; 3.63)	1.80 (0.93; 3.50)
**Intermediate endpoints**				
Change in HbA^{1c}, mmol/mol<br>%	1.8 (1.5; 2.1)<br>0.23 (0.19; 0.26)	1.8 (1.5; 2.2)<br>0.23 (0.19; 0.27)	1.8 (1.4; 2.1)<br>0.22 (0.18; 0.26)	1.8 (1.5; 2.2)<br>0.23 (0.19; 0.28)
Change in body weight, kg	-1.26 (-1.98; -0.54)	-1.26 (-1.98; -0.54)	-1.35 (-2.16; -0.54)	-1.50 (-2.31; -0.68)
Change in systolic blood pressure, mm Hg	-1.11 (-1.76; -0.45)	-1.21 (-1.92; -0.50)	-0.95 (-1.68; -0.22)	-0.97 (-1.75; -0.20)
Change in diastolic blood pressure, mm Hg	-0.62 (-0.98; -0.27)	-0.64 (-1.02; -0.27)	-0.56 (-0.95; -0.16)	-0.60 (-1.01; -0.18)

Abbreviations: ITT, intention-to-treat. OT, on-treatment. CKD, chronic kidney disease. UACR, urinary albumin excretion rate. HR, hazard ratio

ESM Table 3. Characteristics of the population without baseline CKD. Characteristics of patients in the first imputed dataset. Absolute standardized mean difference (SMD) is shown for each variable as a measure of between-group balance.

	Before PSM			After PSM		
	**SGLT2i**	**GLP-1RA**	**SMD**	**SGLT2i**	**GLP-1RA**	**SMD**
**Number**	11421	4594		4430	4430	
**Demographics**						
Sex male, %	6981 (61.1)	2647 (57.6)	0.07	2605 (58.8)	2572 (58.1)	0.02
Age, years	61.0 (8.9)	59.9 (9.5)	0.11	60.2 (9.1)	60.0 (9.5)	0.02
Diabetes duration, years	11.4 (8.5)	9.4 (7.7)	0.24	9.4 (7.6)	9.4 (7.7)	<0.01
**Anthropometrics**						
Weight, kg	88.1 (18.0)	93.8 (18.9)	0.32	92.2 (18.9)	92.9 (18.0)	0.04
Height, cm	167.2 (9.8)	167.5 (9.9)	0.03	167.5 (9.8)	167.5 (9.9)	<0.01
Body mass index, kg/m2	31.5 (5.8)	33.4 (6.2)	0.34	32.8 (6.2)	33.1 (5.8)	0.05
Waist, cm	108.4 (13.2)	112.3 (13.6)	0.29	110.9 (14.1)	111.4 (12.8)	0.04
**Risk factors and laboratory**						
Systolic blood pressure, mm Hg	137.2 (18.7)	136.7 (17.9)	0.03	136.4 (18.6)	136.5 (17.8)	<0.01
Diastolic blood pressure, mm Hg	79.1 (10.0)	80.1 (10.0)	0.09	80.1 (10.1)	80.0 (10.0)	<0.01
Fasting plasma glucose, mg/dl	171.5 (58.0)	162.2 (49.4)	0.17	162.3 (50.5)	162.4 (49.6)	<0.01
HbA1c, mmol/mol<br>%	67.0 (12.1)<br>8.3 (1.5)	64.0 (9.6)<br>8.0 (1.2)	0.26	64.0 (10.4)<br>8.0 (1.3)	64.0 (9.6)<br>8.0 (1.2)	<0.01
Total cholesterol, mmol/l<br>mg/dl	4.6 (1.1)<br>173.1 (43.6)	4.6 (1.2)<br>173.7 (43.9)	0.01	4.6 (1.1)<br>173.4 (42.4)	4.6 (1.2)<br>173.8 (43.9)	<0.01
HDL cholesterol, mmol/l<br>mg/dl	1.2 (0.4)<br>47.1 (14.1)	1.2 (0.4)<br>47.3 (14.1)	0.01	1.2 (0.4)<br>47.4 (13.7)	1.2 (0.4)<br>47.3 (14.1)	<0.01
LDL cholesterol, mg/dl	2.5 (1.0)<br>94.7 (36.9)	2.5 (1.0)<br>96.3 (36.7)	0.04	2.5 (0.9)<br>95.4 (36.0)	2.5 (1.0)<br>96.3 (36.7)	0.02
Triglycerides, mmol/l<br>mg/dl	1.8 (1.4)<br>162.8 (126.2)	1.8 (1.4)<br>156.7 (124.9)	0.05	1.8 (1.2)<br>158.0 (108.9)	1.8 (1.4)<br>157.1 (126.4)	<0.01
eGFR (ml/min/1.73 m^2)	89.1 (14.0)	89.9 (14.3)	0.06	89.7 (14.1)	89.9 (14.3)	0.01
Albumin excretion rate, mg/g	10.4 (7.4)	10.3 (7.3)	<0.01	10.2 (7.3)	10.3 (7.3)	0.01
eGFR slope (ml/min/1.73 m^2/year)	-0.5 (25.8)	0.0 (24.0)	0.02	0.3 (16.3)	-0.1 (23.9)	0.02
**Complications**						
Chronic kidney disease, %	0 (0.0)	0 (0.0)	0.02	0 (0.0)	0 (0.0)	<0.01
Pathologic albuminuria, %	0 (0.0)	0 (0.0)	<0.01	0 (0.0)	0 (0.0)	<0.01
Diabetic Retinopathy; %	1920 (16.8)	475 (10.3)	0.18	472 (10.7)	460 (10.4)	<0.01
Diabetic macular edema, %	258 (2.3)	58 (1.3)	0.07	61 (1.4)	57 (1.3)	<0.01
Stroke / TIA; %	161 (1.4)	48 (1.0)	0.03	51 (1.2)	46 (1.0)	0.01
Carotid atherosclerosis, %	2102 (18.4)	794 (17.3)	0.03	724 (16.3)	771 (17.4)	0.03
Ischemic heart disease; %	1559 (13.7)	404 (8.8)	0.15	424 (9.6)	402 (9.1)	0.02
Left ventricular hypertrophy, %	794 (7.0)	281 (6.1)	0.03	272 (6.1)	269 (6.1)	<0.01
Heart failure, %	346 (3.0)	80 (1.7)	0.08	101 (2.3)	80 (1.8)	0.03
Any site revascolarization, %	1116 (9.8)	308 (6.7)	0.11	324 (7.3)	308 (7.0)	0.01
Microvascular complications, %	2504 (21.9)	647 (14.1)	0.20	646 (14.6)	627 (14.2)	0.01
Macrovascular complications, %	3812 (33.4)	1276 (27.8)	0.12	1264 (28.5)	1244 (28.1)	0.01
Established CVD, %	1863 (16.3)	507 (11.0)	0.15	530 (12.0)	503 (11.4)	0.02
**Glucose lowering medications**						
Metformin, %	9227 (80.8)	3896 (84.8)	0.10	3796 (85.7)	3771 (85.1)	0.02
Sulphonylurea / repaglinide, %	731 (6.4)	553 (12.0)	0.21	480 (10.8)	507 (11.4)	0.02

Pioglitazone, %	195 (1.7)	237 (5.2)	0.21	149 (3.4)	180 (4.1)	0.04
Acarbose, %	69 (0.6)	22 (0.5)	0.02	23 (0.5)	22 (0.5)	<0.01
Bolus insulin, %	3095 (27.1)	98 (2.1)	0.65	98 (2.2)	98 (2.2)	<0.01
Basal insulin, %	4728 (41.4)	944 (20.5)	0.44	930 (21.0)	921 (20.8)	<0.01
**Other medications**						
Statins, %	6353 (55.6)	2308 (50.2)	0.11	2244 (50.7)	2248 (50.7)	<0.01
Anti-platelet agents, %	4402 (38.5)	1485 (32.3)	0.13	1465 (33.1)	1450 (32.7)	<0.01
RAS blockers, %	6381 (55.9)	2537 (55.2)	0.01	2433 (54.9)	2444 (55.2)	<0.01
Beta blockers, %	3281 (28.7)	1182 (25.7)	0.07	1174 (26.5)	1158 (26.1)	<0.01
Calcium channel inhibitors, %	2199 (19.3)	916 (19.9)	0.02	905 (20.4)	877 (19.8)	0.02
Diuretics, %	3058 (26.8)	1369 (29.8)	0.07	1273 (28.7)	1299 (29.3)	0.01
Anticoagulants, %	247 (2.2)	84 (1.8)	0.02	87 (2.0)	81 (1.8)	<0.01

Abbreviations: eGFR, estimated glomerular filtration rate according to the CKD-EPI equation. CKD, chronic kidney disease. TIA, transient ischemic attack. CVD cardiovascular disease. RAS, renin angiotensin system.

ESM Figure 1. Change in albuminuria in the primary ITT analysis. a) Change from baseline in albuminuria on the continuous scale. Numbers below indicate patients who contributed with values at each time point. b) Kaplan-Meier curves for new-onset macroalbuminuria in the two groups. Numbers below indicate patients at risk. *observation closed at 54 months.

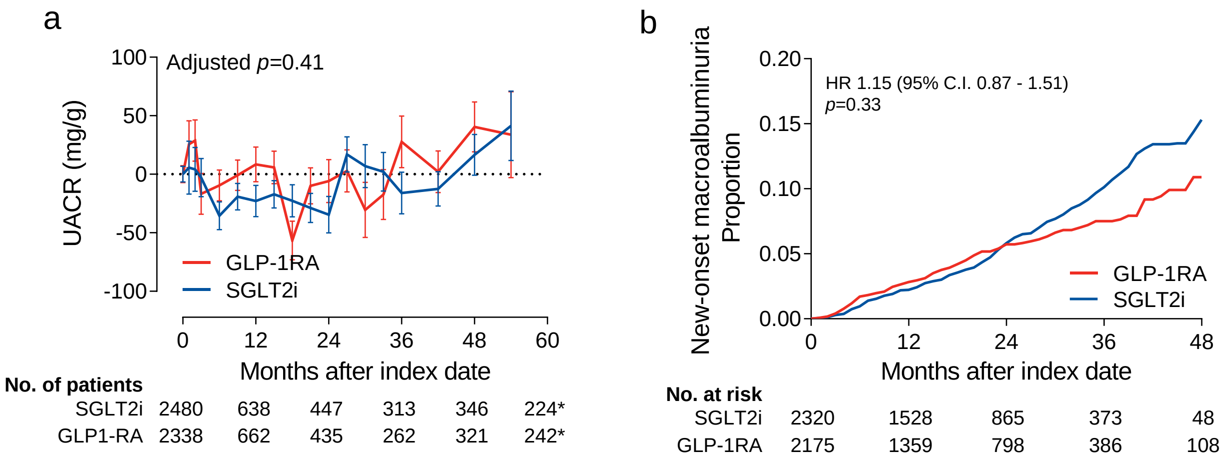

ESM Figure 2. Categorical outcomes in the OT population. The forest plot illustrates the most important outcomes in the on-treatment (OT) population. Tabular results are presented as crude rates in each group, hazard ratios (HR) and 95% confidence intervals (C.I.) along with their p-values.

Endpoint	Crude rates / 1000 PYE SGLT2i	GLP.1RA	Adjusted hazard ratio (95% C.I.)	p-value
New-onset CKD	25.7	31.3	0.83 (0.69 - 0.99)	0.041
Worsening CKD class	240.2	260.7	0.93 (0.86 - 0.99)	0.037
Sustained >40% eGFR reduction	15.0	18.2	0.83 (0.62 - 1.11)	0.217
Sustained >57% eGFR reduction	4.4	6.7	0.65 (0.39 - 1.09)	0.110
New-onset macroalbuminuria	23.8	23.0	1.05 (0.76 - 1.45)	0.749

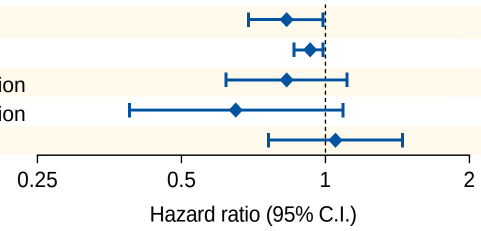

ESM Figure 3. Main results in the population without baseline CKD. a) Change in eGFR (primary outcome) in the two groups. b) Kaplan-Meier curve for the worsening of CKD class. c) Change in albuminuria in the two groups. d) Kaplan-Meier curve for new-onset macroalbuminuria. In a) and c), the table below shows the number of patients contributing with values to the model. In b) and d), the table below shows the number of patients at risk. e) Forest plot of kidney outcomes. Tabular results are presented as crude rates in each group, hazard ratios (HR) and 95% confidence intervals (C.I.) along with their p-values.

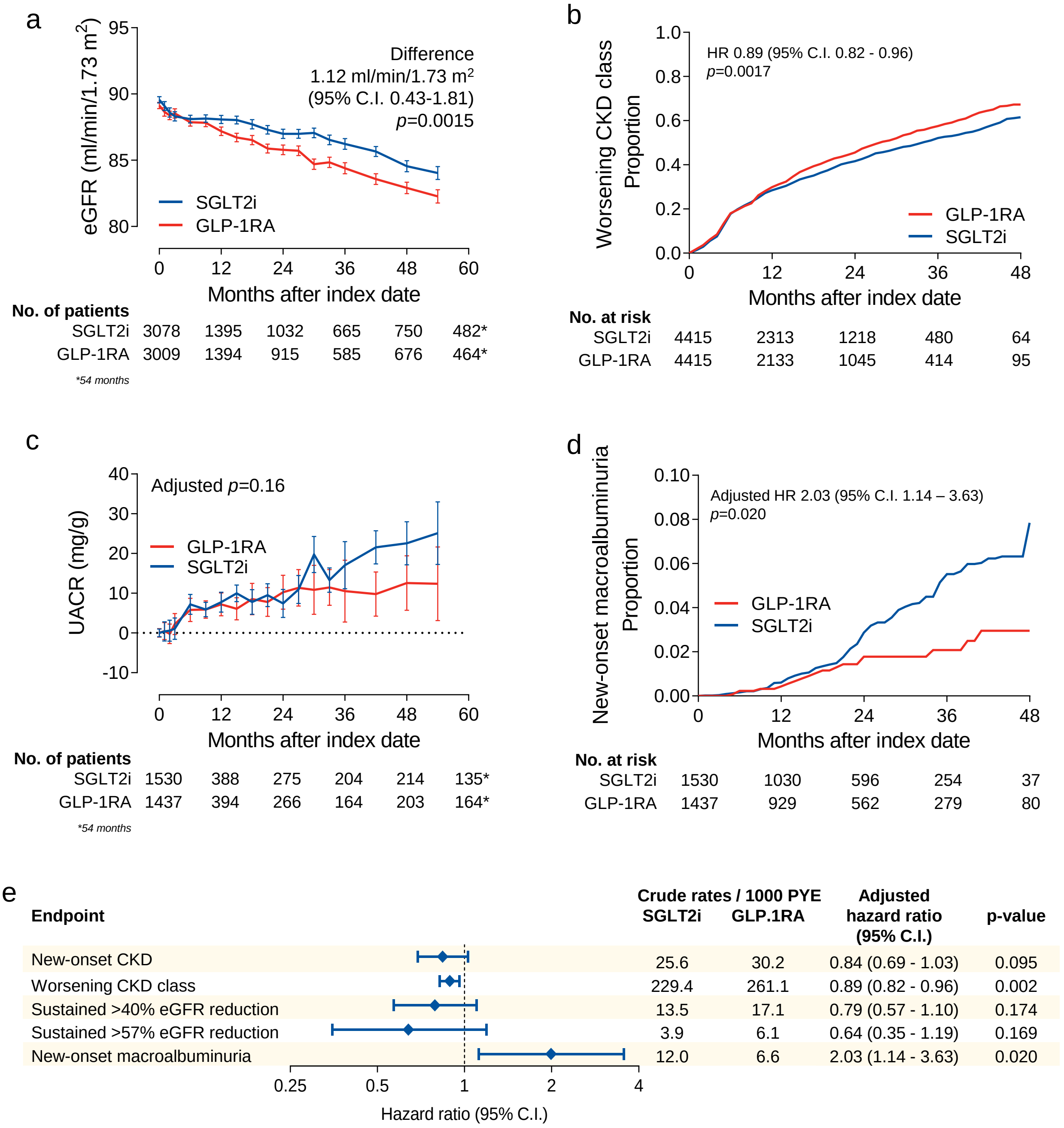

Supplement: Supplementary file 1 — ESM (PDF 464 KB) [file 125_2024_6251_MOESM1_ESM.pdf]
